# Bioremediation of Chlorpyrifos Contaminated Soil: Microcosm Investigation and 16S rRNA Gene Amplicon Sequencing

**DOI:** 10.1111/1758-2229.70457

**Published:** 2026-11-09

**Authors:** Le Thi Nhi‐Cong, Vu Duc Toan, Dang Viet Lam, Tran Thi Mai, Cung Thi Ngoc Mai, Bui Thi Kim Anh, Dong Diem Linh

**Affiliations:** ^1^ Institute of Biology, Vietnam Academy of Science and Technology Hanoi Vietnam; ^2^ Graduate University of Science and Technology, Vietnam Academy of Science and Technology Hanoi Vietnam; ^3^ ROOM Strong Research, Environmental and Life Science Research Laboratory, Thuyloi University Hanoi Vietnam; ^4^ Chemical and Environmental Laboratory, Quality Assurance and Testing Center 1, Directorate for Standards and Quality, Ministry of Science and Technology Hanoi Vietnam; ^5^ Institute of Science and Technology for Energy and Environment, Vietnam Academy of Science and Technology Hanoi Vietnam; ^6^ University of Science and Technology of Hanoi, Vietnam Academy of Science and Technology Hanoi Vietnam

**Keywords:** *Bacillus toyonensis*, biodegradation, biofilm formation, pesticide, sugarcane bagasse

## Abstract

This study describes the isolation and characterization of two biofilm‐forming bacterial strains, *Pseudomonas aeruginosa* MD3 and *Bacillus toyonensis* LD07, capable of degrading chlorpyrifos. These isolates demonstrated remarkable phenotypic plasticity, maintaining biofilm formation across varying pH, temperature and salinity levels. To maximize microbial stability and metabolic efficiency, sugarcane bagasse was utilized as a support matrix. Analytical characterization (EDX, BET and XRD) confirmed that the bagasse possesses a highly porous architecture, serving as a protective scaffold and an auxiliary carbon source for synergistic co‐metabolism. A bio‐product formulated via solid‐state fermentation on this matrix exhibited exceptional bioremediation efficacy, eliminating 99% of chlorpyrifos at an extreme concentration of 4500 ppm within 35 days. Furthermore, 16S rRNA gene amplicon sequencing of coffee‐growing soils across different treatments (natural control, pesticide‐stressed and 10% bio‐product amended) with accession numbers PRJNA1529359 revealed distinct shifts in microbial community dynamics. While the taxonomic landscape was broadly dominated by Bacillota, Pseudomonadota and Actinomycetota, the bio‐product amendment caused *P. aeruginosa* and *B. toyonensis* to become the dominant taxa. Ultimately, these insights underscore the viability of cost‐effective agricultural by‐products for cell immobilization and highlight metagenomics as a powerful predictive framework for monitoring the remediation of anthropogenically stressed ecosystems.

## Graphical abstract

This study characterized biofilm‐forming bacterial strains *Pseudomonas aeruginosa* MD3 and *Bacillus toyonensis* LD07 immobilized on sugarcane bagasse. This highly porous bio‐product demonstrated exceptional phenotypic plasticity and robust bioremediation efficacy, achieving 99% chlorpyrifos degradation at 4500 ppm. Metagenomic analysis confirmed significant ecological shifts, validating this system for xenobiotic remediation.

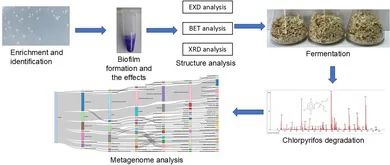

## 1 Introduction

Contemporary agriculture has become increasingly reliant on pesticides, which serve as the primary strategy for managing crop pests and diseases, thereby substantially enhancing yields. Global pesticide consumption has escalated to approximately 3.5 billion kg annually, with a significant proportion deemed excessive or superfluous in numerous countries (Eyhorn et al. 2015). Although designed to safeguard crops, pesticides exert deleterious effects on ecosystems. Their widespread application imperils human and animal health owing to inherent high toxicity and environmental persistence (Varghese et al. 2022).

Chlorpyrifos [O,O‐diethyl O‐(3,5,6‐trichloro‐2‐pyridyl) phosphorothioate] (CP) is a widely applied organophosphate pesticide used to manage chewing and sucking insect pests as well as mites in the cultivation of economically significant crops, including rice, citrus fruits, vegetables and coffee (Racke 1993; Thengodkar and Sivakami 2010). Due to their extensive use, chlorpyrifos and related compounds are the predominant organophosphorus pesticides in the United States and other regions (USEPA 2011). Nevertheless, prolonged agricultural and horticultural use has led to widespread environmental contamination, posing substantial risks to ecosystems and human health owing to their inherent toxicity and recalcitrance to natural degradation processes. Elevated CP concentrations markedly suppress brain cholinesterase (ChE) activity, impairing nervous, respiratory and cardiovascular functions. Although ambient environmental levels are typically low and insufficient to cause pronounced ChE inhibition, they can selectively disrupt fatty acid amide hydrolase activity, adversely impacting neurological function and behaviour (Farag et al. 2003; Sinha et al. 2006; Carr et al. 2014). Consequently, remediation of chlorpyrifos contamination remains a pressing global challenge (Liu et al. 2016).

A variety of approaches have been developed for the detoxification of chlorpyrifos‐contaminated wastewater and soils. Physicochemical strategies, including photodegradation, metal nanoparticle remediation and UV/H_2_O_2_ advanced oxidation processes, have been extensively investigated (Affam and Chaudhuri 2013). However, most of these methods remain unimplemented due to their inefficacy or prohibitive costs (Raj and Kumar 2022). In contrast, microbial biodegradation offers a promising alternative for remediating chlorpyrifos‐polluted environments, attributed to its superior degradative efficiency, cost‐effectiveness and environmental compatibility (Abraham et al. 2014; Varghese et al. 2022). A wide range of CP‐degrading bacterial strains, such as *Bacillus safensis* FO‐36bT (Abdelbagi et al. 2018), *Cupriavidus nantongensis* X1^T^ (Fang et al. 2019), *Klebsiella* sp. S6 (John et al. 2018), *Pseudomonas indica* PKDB2 (Aswani et al. 2018), *Pseudomonas putida* MB285 (Liu et al. 2016), etc., have been reported, highlighting the potential of microbial remediation for contaminated soil. In 2020, Govarthanan et al. (2020) isolated the bacterium *Shewanella* sp. BT05 which could remove 93% CPs with the initial concentration of 50 ppm in the presence of 3.5% glucose in pH 7.0 at 32.5°C. Ahir et al. (2020) reported a novel isolate *Tistrella* sp. AUC10 which could degrade 99.86% CPs with the initial concentration of 2 ppm after 20 days. Hadibarata et al. (2023) isolated a bacterial strain *Hortaea* sp. B15 which could degrade 89.5% CPs with the initial concentration of 100 ppm. Wepukhulu et al. (2024) isolated seven bacterial strains exhibiting CP‐degrading potential which were characterized and identified as *Alcaligenes faecalis* , *Bacillus weihenstephanensis* , *Bacillus toyonensis*, *Alcaligenes* sp. strain *SCAU23*, *Pseudomonas* sp. strain *PB845W*, *Brevundimonas diminuta* and *uncultured bacterium* clone 99. Ghorab et al. (2026) reviewed many species, including *Pseudomonas, Bacillus, Acinetobacter and Aspergillus*, that can catabolize a broad repertoire of OP compounds, such as CPs, in both soil and aqueous matrices.

Among microbial remediation strategies, biofilm‐based systems have attracted increasing attention because immobilized cells generally tolerate environmental stress better than planktonic cells and often exhibit greater stability during long‐term biodegradation. Immobilization also helps maintain high cell density and prolonged metabolic activity, improving the degradation of persistent organic pollutants (Biswal and Malik 2022; Sridharan and Krishnaswamy 2022; Nadar and Khan 2024). Therefore, selecting an appropriate carrier material is critical for developing an efficient bioaugmentation product (Jagaba et al. 2024). Numerous materials were successfully applied in persistent component removal such as biochar, coconut fibre, cotton, … (Toledo‐Jaldin et al. 2020; Nga et al. 2024; Nhi‐Cong et al. 2020; Al‐Amshawee et al. 2020). Among, sugarcane bagasse—a by‐product of the sugar industry—comprises 40%–50% cellulose, 25%–30% hemicellulose and 20%–25% lignin (Nunes et al. 2020; Hassan et al. 2024). Sugarcane bagasse could enhance immobilization efficiency of bacteria to remove dye, textile dye and other hazardous materials (Petpheng et al. 2025; Gita et al. 2021; Sarker et al. 2017; Basak et al. 2019). These sugars function as critical carbon sources for polyhydroxyalkanoate‐producing bacteria, facilitating efficient biosynthesis under nutrient‐limited conditions (Zheng et al. 2020). Therefore, utilizing sugarcane bagasse to attach microorganisms and apply it in CP‐treatment will support a habitat and the first carbon source for them, which is called the co‐metabolism method. Co‐metabolism is an effective strategy for the biodegradation of recalcitrant organic compounds (Lu et al. 2016). It enhances the biodegradation of persistent pollutants that are poorly degradable by bacteria when provided as the sole carbon source, owing to the action of corresponding enzymes, cofactors and the synergy among diverse dominant functional microorganisms (Zhou et al. 2019). Numerous studies have demonstrated that low‐molecular‐weight polycyclic aromatic hydrocarbons (PAHs; fewer than four fused benzene rings) can serve as co‐substrates to improve the biodegradability of high‐molecular‐weight PAHs. Wang et al. (2025) observed that supplementation with naphthalene (10 mg/L) increased the pyrene (1 mg/L) removal efficiency of *Ochrobactrum* sp. MB‐2 from 15.3% to 41%. Zhou et al. (2019) reported that the removal efficiencies of phenanthrene and pyrene in a phenanthrene‐pyrene mixture were consistently higher than those in single‐substrate experiments.

Recent advances in 16S rRNA gene amplicon sequencing have provided valuable insights into the composition and dynamics of microbial communities involved in bioremediation. Beyond identifying individual degraders, this approach enables the evaluation of microbial community responses to environmental contaminants and bioaugmentation strategies, thereby improving our understanding of the ecological processes underlying pesticide degradation. Three bacterial species exhibiting monocrotophos‐degrading capacity were functionally characterized (Borchetia et al. 2018).

Although numerous chlorpyrifos‐degrading bacteria have been isolated, relatively few studies have combined microbial isolation, biofilm immobilization and microbial community analysis to evaluate the performance of bioaugmentation systems under soil conditions. In particular, information on using agricultural by‐products as microbial carriers and their influence on indigenous bacterial communities during chlorpyrifos remediation remains limited.

In this study, two chlorpyrifos‐degrading bacterial strains were isolated from coffee‐growing soil in Lam Dong province, Vietnam and evaluated for their biofilm‐forming ability under different medium conditions. Sugarcane bagasse was investigated as a carrier for bacterial immobilization to develop a bioaugmentation product for chlorpyrifos‐contaminated soil. In addition, 16S rRNA gene amplicon sequencing was used to examine changes in the soil bacterial community following bioaugmentation. This integrated approach provides insights into both the remediation performance of the bio‐product and its influence on the resident microbial community.

## 2 Materials and Methods

### 2.1 Materials

In this study, soil samples (10–30 cm depth) were collected from coffee‐growing soil in Lam Ha ward, Lam Dong province (120°07.467 E, 23°02.173 N) and stored at ambient temperature.

Chlorpyrifos was purchased from Accustandard and filter‐sterilized using a 0.2 μm syringe filter. All other chemicals were purchased from Sigma‐Aldrich Co.

Mineral salt medium (MSM) containing (g/L): K_2_HPO_4_ 1.8, NH_4_CI 4.0, NaCl 0.1, MgSO_4_.7H_2_O 0.2, FeSO_4_.7H_2_O 0.01; pH 7.2 ± 0.2.

Nutrient Broth (NB) medium (g/L): peptone 10, beef extract 3–5 g, NaCl 5; pH 7.4 ± 0.2.

### 2.2 Methods

#### 2.2.1 Isolation and Identification

##### 2.2.1.1 Enrichment and Isolation

One gram of each sediment sample was incubated in 100 mL of MSM supplemented with 50 ppm chlorpyrifos and 10% glucose as a co‐substrate. The incubation was shaken at 150 rpm at 25°C–30°C for 7 days. After 7 days, 10 mL of the bacterial shaking‐cultivation was then transferred to the next 90 mL MSM provided with 50 ppm CP and 10% glucose to enrich the utilized chlorpyrifos organisms. After the next 7 days, the culture was repeated. After 3 rounds of enrichment, the inoculum was spread onto Petri plates and incubated at 28°C ± 2°C for 24–48 h (Wang et al. 2019). The number of colonies on the plates was enumerated to quantify bacterial abundance. Colonies exhibiting distinctive morphological characteristics were subsequently streaked onto fresh plates for isolation. Purified isolates were preserved on slants at 4°C.

##### 2.2.1.2 Identification

###### 2.2.1.2.1 DNA Preparation

Total bacterial genomic DNA was extracted according to Zhou et al. (1996), with minor modifications. Prepare 1 L of 1 M NaPP by mixing 53 mL of 1 M NaH_2_PO_4_ with 947 mL of 1 M Na_2_HPO_4_ and adjusting the pH to 8.0. Bacterial biomass was harvested from overnight cultures on LB medium by centrifugation at 7000 × *g* for 10 min at 4°C. The pellet was resuspended in 540 μL of extraction buffer (1 M Tris–Cl, 0.5 M EDTA, 1 M NaPP, 1 M NaCl and 1% CTAB) and vortexed thoroughly. Subsequently, 20 μL of proteinase K (20 mg/mL) was added and the suspension was incubated with shaking at 37°C for 30 min, followed by the addition of 60 μL of 20% SDS and incubation at 65°C for 2 h. Thereafter, 600 μL of chloroform: isoamyl alcohol (24:1, CI) was added, the tubes were mixed by gentle inversion and centrifuged at 12,000 × *g* for 15 min. The aqueous phase was carefully transferred to a new Eppendorf tube. DNA was precipitated by adding isopropanol (six volumes of the aqueous phase) and incubating at room temperature for 1 h. The DNA pellet was recovered by centrifugation at 12,000 × *g* for 15 min at 4°C, washed with 500 μL of cold 70% ethanol and centrifuged again at 12,000 × *g* for 10 min at 4°C. The supernatant was discarded and the pellet was air‐dried briefly before resuspension in 25–30 μL of sterile deionized water. The DNA was kept at −20°C until use.

###### 2.2.1.2.2 Standard PCR

The selected strains were identified by 16S rRNA analysis as described by Ahirwar et al. (2019) with modification. In detail, genomic DNA was extracted and the 16S rRNA gene target region was amplified using DreamTaq DNA Polymerase (Thermo Fisher Scientific, Waltham, MA, USA). The DNA extract was used as template for PCR with the primers 27F (forward: 5′‐AGAGTTTGATCCTGGCTCAG‐3′) and 1492R (reverse: 5′‐GGTTACCTTGTTACGACTT‐3′) (Miller et al. 2013). The reaction mixture included master mix 10 μL, forward and reverse primer 0.1 μL, taq polymerase 0.2 μL and 39.6 μL water was added and volume of reaction made 50 μL. The PCR was carried out for 35 cycles of denaturation at 94°C for 60 s, annealing at 55°C for 45 s and extension at 72°C for 60 s in a Gene Amp PCR System 9700 thermalcycler.

###### 2.2.1.2.3 Sequencing Procedure

Purified PCR products, obtained using the AccuPrep PCR Purification Kit (Bioneer, Korea), were employed as templates for sequencing reactions carried out on an ABI PRISM 3100 Avant Genetic Analyser (Applied Biosystems, USA). Raw nucleotide sequence data were processed using ABI PRISM 3100 Avant Data Collection software (version 1.0).

###### 2.2.1.2.4 Bioinformatic Analysis

Sequence similarity searches were conducted against the NCBI nucleotide database using the BLASTN algorithm. Subsequent sequence analyses, including multiple sequence alignment and phylogenetic tree construction, were performed using BioEdit, CLUSTAL X and MEGA11 software packages. The obtained gene sequences were deposited in the GenBank database under assigned accession numbers.

#### 2.2.2 Biofilm Formation and Effects by pH, Temperature, NaCl Concentration

Biofilm formation tests were performed using the method described by Shimada et al. (2012) with minor modifications. Specifically, overnight cultures were adjusted to an OD600 of 0.3, then inoculated at 1% (v/v) into 300 μL of NB medium in 1.5 mL microcentrifuge tubes (TC131615, Nippon Genetics, Tokyo, Japan). The tubes were incubated statically at 30°C ± 2°C for 24–48 h. Subsequently, the pellicles and medium were removed and the tubes were gently rinsed with distilled water, then filled with 500 μL of 1% crystal violet (CV) solution. After 25 min, the CV solution was discarded and the tubes were washed again with distilled water. The CV bound to the biofilm was extracted with 400 μL of 33% acetic acid and quantified by measuring absorbance at 570 nm. NB medium was used to assess bacterial biofilm formation capacity, with *Acinetobacter calcoaceticus* P23 (Yamaga et al. 2010) as the positive control and a tube without inoculum as the negative control. Each experiment was conducted in triplicate.

##### 2.2.2.1 Effect of pH

To elucidate the effect of pH on biofilm formation, the NB medium was adjusted to pH 4, 5, 6, 7, 8 and 9. After that, a biofilm formation procedure similar to that described above was carried out.

##### 2.2.2.2 Effect of Temperature

The suitable pH value was used in further experiments. The bacteria were cultivated in a medium at that pH and incubated at different temperatures, including 4°C, 20°C, 25°C, 30°C, 35°C and 45°C. The obtained results indicated the effect of temperature on biofilm formation of the strains.

##### 2.2.2.3 Effect of NaCl Concentration

The optimal pH and temperature values were then used in the next experiments. The effect of NaCl concentration on biofilm formation was evaluated by varying the NaCl concentration in the NB medium components to 0%, 5%, 10% and 15%. After preparing NB medium at the specified NaCl concentrations with appropriate pH, the media were sterilized and used to culture bacteria. The cultures were stored at the specified temperature to assess biofilm‐forming capacity and OD600 values were measured as described above.

#### 2.2.3 Physicochemical Characteristics of Sugarcane Bagasse

##### 2.2.3.1 The Elemental Composition Analysis by Using Energy‐Dispersive X‐Ray (EDX) Approach

Measurements were conducted on a HORIBA 7593‐H/HITACHI S‐4800 instrument, with a working distance of 15 mm and an accelerating voltage of 20 kV. The procedure was introduced in Instruction manual for model S‐4800 field emission scanning electron microscope (https://www.scu.edu/media/school‐of‐engineering/photos/cns/SEM‐Hitachi‐S‐4800‐Instruction‐Manual.pdf). Samples were mounted in the HITACHI S‐4800 scanning electron microscope (SEM), coated with platinum for 40 s and analysed at 5 kV with a tip‐to‐sample distance of approximately 5–6 mm. The sample was excited by a high‐energy electron beam (5 kV) and the EDX detector captured the characteristic X‐rays emitted from the sample. The energy and intensity of these X‐rays were analysed using software to identify the elemental types and their relative contents in the sample.

##### 2.2.3.2 The Brunauer–Emmett–Teller (BET) Isotherm Method

The specific surface area can be determined from the BET adsorption isotherm (Bae et al. 2010). This involves physically adsorbing nitrogen gas (N_2_) onto the sample at the boiling point of liquid nitrogen (77.4 K), measuring the adsorbed gas volume or mass at various pressures, plotting the adsorption isotherm and applying the BET equation in Excel to calculate the specific surface area.

###### 2.2.3.2.1 Sample Preparation

The bagasse sample was cleaned by using distilled water and dried under vacuum to remove surface impurities and moisture, ensuring accurate measurements. Measurements were performed using the Horiba SA‐9600 instrument (Japan).

###### 2.2.3.2.2 Degassing

Dinitrogen (N_2_) was employed as the molecular adsorbate (77.4 K). The analysis proceeded through a systematic, incremental increase in relative pressure (*P*/*P*_0_), during which the volume of gas adsorbed onto the sample surface was precisely quantified to construct the adsorption isotherm. Subsequently, the desorption branch was recorded by progressively reducing the system pressure. This complete adsorption–desorption cycle facilitates the identification of hysteresis loops, which are critical for elucidating the pore geometry and total specific surface area of the biomass‐derived carrier.

##### 2.2.3.3 The Material Structure Was Characterized Using X‐Ray Diffraction (XRD) Technique

The XRD measurements were performed using a D8 Advance diffractometer (Bruker, Germany) (https://fr.scribd.com/document/874176850/Xrd‐d8‐Advance‐Bb‐Gid). Spectral analysis was conducted with Diffrac eva Software (Bruker).

##### 2.2.3.4 Scanning Electron Microscopy (SEM) Images

The formed biofilms and sugarcane bagasse were gently placed onto poly‐l‐lysine‐coated glass slides. The samples were then fixed with glutaraldehyde and OsO_4_, dehydrated using solvents containing ethanol, isoamyl acetate and CO_2_ to remove residual water from the biofilms and bagasse and subsequently sputter‐coated with platinum for observation under a HITACHI S‐4800 scanning electron microscope (Nhi‐Cong et al. 2020).

#### 2.2.4 Fermentation to Form a Bio‐Product

Sugarcane bagasse was pre‐treated as described by Alessandrello et al. (2017) with minor modifications. Briefly, the bagasse was split into pieces of approximately 0.5–1 mm, then it was sterilized by autoclaving at 121°C for 15 min.

The bio‐product was prepared following the method of Nhi‐Cong et al. (2020) with minor modifications. Equal volumes of the two bacterial strains were cultivated in nutrient broth (NB). Cells were harvested, washed with phosphate buffer and resuspended in fresh NB medium to obtain a cell density of approximately 10^7^–10^8^ CFU mL^−1^. The bacterial suspension was then inoculated into 100 mL NB medium containing 5 g of sterile sugarcane bagasse and incubated statically at 30°C for 48 h to allow biofilm formation on the carrier surface. The immobilized bio‐product was either used immediately or stored at 4°C for no longer than 3 weeks before use.

#### 2.2.5 Chlorpyrifos Degradation

To evaluate CP degradation capacity of the bio‐product, 460, 575, 1000 and 4500 ppm CP were provided to 1 kg normal soil then mix with 1; 5; 10% or 25% bio‐product and store them in a box with a cover (Table 1). The experiments were conducted as described by Nhi‐Cong et al. (2020) with modification. The controls were divided into 11 groups including E1, only natural coffee‐growing soil; E2, that soil supplemented with 459.56 ± 5 ppm CP; E3, that soil supplemented with 459.56 ± 6 ppm CP and 10% of sterilized sugarcane bagasse; E4, that soil supplemented with 459.55 ± 10 ppm CP and bacteria; E9, E16 and E23 were prepared in a similar manner to E2 but differed in the amount of CP such as 563.47 ± 9, 994.13 ± 14 and 4389.31 ± 29 ppm, respectively; E10, E17 and E24 were conducted similarly to E3 but differed in the concentration of substrate; E11, E18 and E25 were similar to E4 but different from the substrate concentrations. The controls E4, E11, E18 and E25 were incubated with 10% of the liquid bacterial culture, which has a density of about 10^7^–10^8^ CFU/mL, without a carrier. After 48 h, the experiments. The samples at 0, 14 and 35 days were evaluated for CP degradation efficiency using GC–MS/SIM. The samples were dried and finely crushed before analysis. 20 g of sample, 10 g of anhydrous Na_2_SO_4_ and surrogate standard were transferred and Soxhlet extraction was performed with a 150 mL mixture of dichloromethane: acetone (1:1, v/v) for 16–24 h. The extract was collected, evaporated and then transferred into *n*‐hexane. The eluate solvent was cleaned through a Florisil column, the column was rinsed with 30 mL *n*‐hexane and the rinse was discarded. 180 mL of a mixture of ethyl ether and n‐hexane was used to elute, evaporated and blown into a N_2_ stream to a final volume of 1 mL. Add internal standard, then analysed by GCMS/SIM (Tadeo et al. 2012; El‐Gohary 2013).

**TABLE 1:** Experimental sign.

	Experiment information	The amount of CP at 0 h	±SD
E1	Control soil	11.84	2
E2	Soil +0.01% CP without carrier and bacteria	459.56	5
E3	Soil +0.01% CP + carrier	459.56	6
E4	Soil +0.01% CP + bacteria	459.55	10
E5	Soil +0.01% CP + 1% bio‐product	459.52	10
E6	Soil +0.01% CP + 5% bio‐product	459.51	12
E7	Soil +0.01% CP + 10% bio‐product	459.50	11
E8	Soil +0.01% CP + 25% bio‐product	459.49	8
E9	Soil +0.05% CP without carrier and bacteria	563.47	9
E10	Soil +0.05% CP + carrier	563.41	9
E11	Soil +0.05% CP + bacteria	563.37	7
E12	Soil +0.05% CP + 1% bio‐product	561.47	8
E13	Soil +0.05% CP + 5% bio‐product	560.58	9
E14	Soil +0.05% CP + 10% bio‐product	561.31	7
E15	Soil +0.05% CP + 25% bio‐product	560.12	13
E16	Soil +0.1% CP without carrier and bacteria	994.13	14
E17	Soil +0.1% CP + carrier	994.12	16
E18	Soil +0.1% CP + bacteria	993.12	20
E19	Soil +0.1% CP + 1% bio‐product	994.25	21
E20	Soil +0.1% CP + 5% bio‐product	994.11	23
E21	Soil +0.1% CP + 10% bio‐product	993.20	20
E22	Soil +0.1% CP + 25% bio‐product	993.05	18
E23	Soil +0.5% CP without carrier and bacteria	4389.31	29
E24	Soil +0.5% CP + carrier	4347.74	30
E25	Soil +0.5% CP + bacteria	4222.44	36
E26	Soil +0.5% CP + 1% bio‐product	4223.43	30
E27	Soil +0.5% CP + 5% bio‐product	4223.43	34
E28	Soil +0.5% CP + 10% bio‐product	4222.43	32
E29	Soil +0.5% CP + 25% bio‐product	4219.63	31

Abbreviation: CP: chlorpyrifos.

#### 2.2.6 16S rRNA Gene Amplicon Sequencing

The samples, including M0, coffee‐growing soil, M1, coffee‐growing soil supplemented with 4500 ppm CP and M3, coffee‐growing soil supplemented with 4500 ppm CP and 10% bio‐product, were analysed using metagenomic techniques after 35 days of cultivation.

##### 2.2.6.1 DNA Extraction

Genomic DNA was extracted using the DNeasy PowerSoil Pro Kit (Qiagen, USA) following the manufacturer's instructions (DNeasy PowerSoil Pro Kit Handbook, 2023).

##### 2.2.6.2 DNA Quality Assessment

DNA concentration was quantified fluorometrically using a Qubit fluorometer. The OD260/OD280 ratio was determined spectrophotometrically. Samples were deemed suitable for downstream experiments if they met the following criteria: concentration ≥ 0.20 ng/μL, total yield ≥ 15.00 ng and OD260/OD280 ≥ 1.50.

##### 2.2.6.3 Library Preparation

Full‐length 16S rDNA amplicons were amplified by PCR using the universal primers 27F and 1492R. Libraries were prepared using the Ligation Sequencing Kit (Oxford Nanopore Technologies, USA) according to the manufacturer's protocol. Library concentration was quantified using the Qubit dsDNA assay (Thermo Fisher Scientific) per the manufacturer's instructions. Quality control (QC) criteria for DNA libraries: concentration ≥ 15.00 ng/μL.

##### 2.2.6.4 Sequencing

Prepared libraries were sequenced on the ONT Flongle platform.

###### 2.2.6.4.1 Alpha—Diversity

The *alpha diversity*, as measured by the Shannon index, of the metagenomic profiles was computed from BIOM‐formatted output tables generated by FOCUS/SUPERFOCUS using the QIIME software package (Caporaso et al. 2010).

#### 2.2.7 Statistical Analysis

One‐way analysis of variance (ANOVA) followed by Fisher's post hoc test was performed on the collected data to identify significant differences (*p* < 0.05) among means derived from triplicate measurements. Analyses were conducted using MINITAB statistical software, version 15 for Windows.

Biofilm formation and chlorpyrifos degradation were reported as means for three separate experiments with replicated batch cultures and the standard deviation was no more than 5%. Excel software 2016 was utilized to calculate the average value, standard deviation (SD) and Figures.

## 3 Results

### 3.1 Isolation and Identification

After three rounds of enrichment in mineral salt medium supplemented with 10% glucose and 50 ppm chlorpyrifos, two bacterial strains, designated MD3 and LD07, were successfully isolated. Based on 16S rRNA gene sequencing, strain MD3 was identified as *Pseudomonas aeruginosa* , while strain LD07 was identified as *Bacillus toyonensis*. The two isolates shared 100% sequence similarity with *Pseudomonas aeruginosa* ATCC 10145 and *Bacillus toyonensis* BCT‐7112, respectively (Figure 1).

**FIGURE 1:**
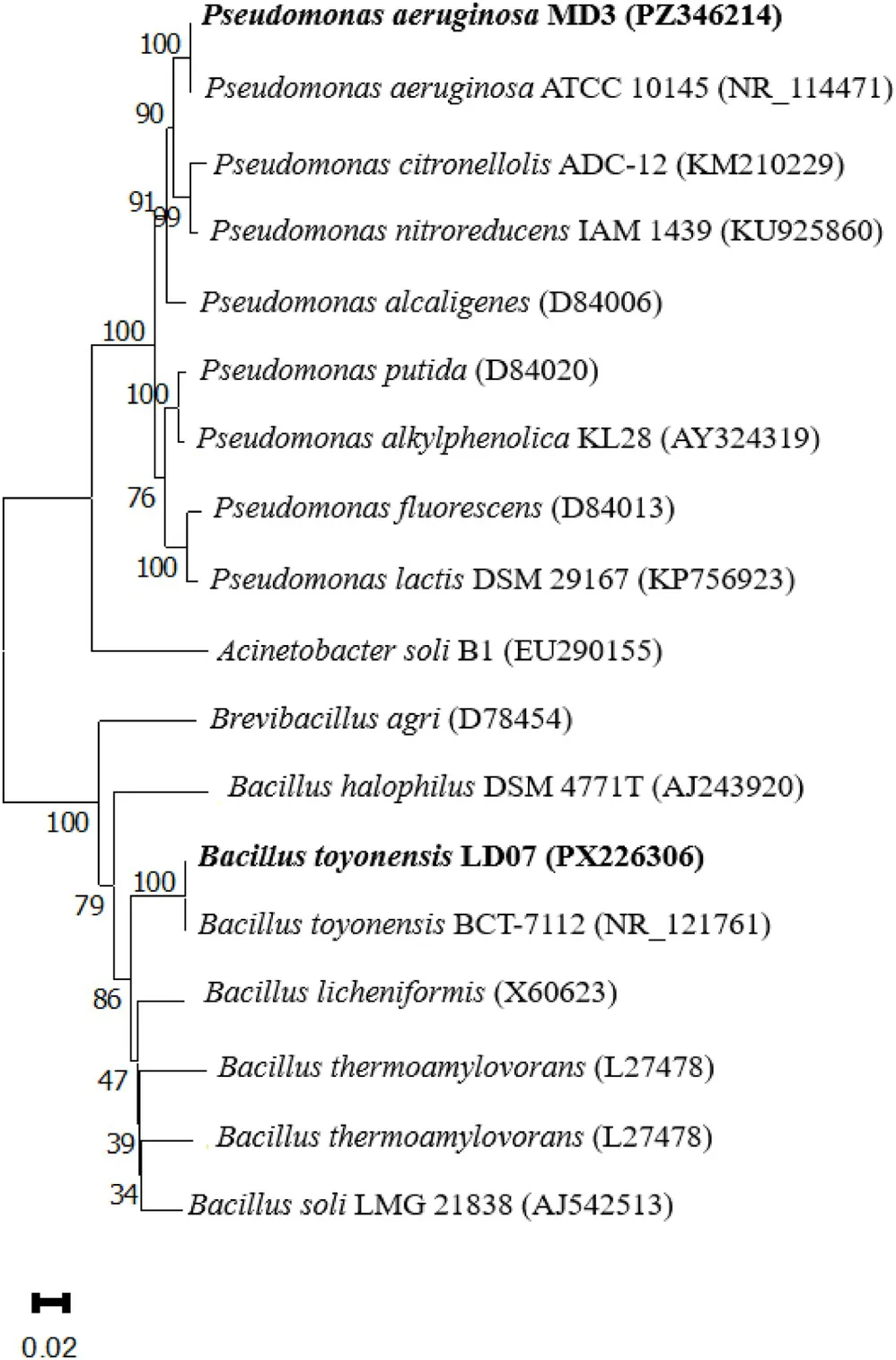
Phylogenetic tree constructed using the neighbour‐joining method based on 16S rRNA gene sequences, illustrating the relationships of strains MD3 and LD07 with reference taxa within the domain Bacteria. Bootstrap values (based on 1000 replicates) are shown at the nodes. The scale bar indicates the number of nucleotide substitutions per site.

### 3.2 Biofilm Formation and Effects by pH, Temperature and NaCl Concentrations

The 2 bacterial strains were evaluated for their biofilm‐forming capacity and the results are shown in Figure 2a. The 2 strains formed biofilms more readily than the positive control, *A. calcoaceticus* P23. No biofilm was observed in the negative control (NB medium only). Then, the effects of pH, temperature and NaCl concentration were conducted and the results were shown in Figure 2b–d.

**FIGURE 2:**
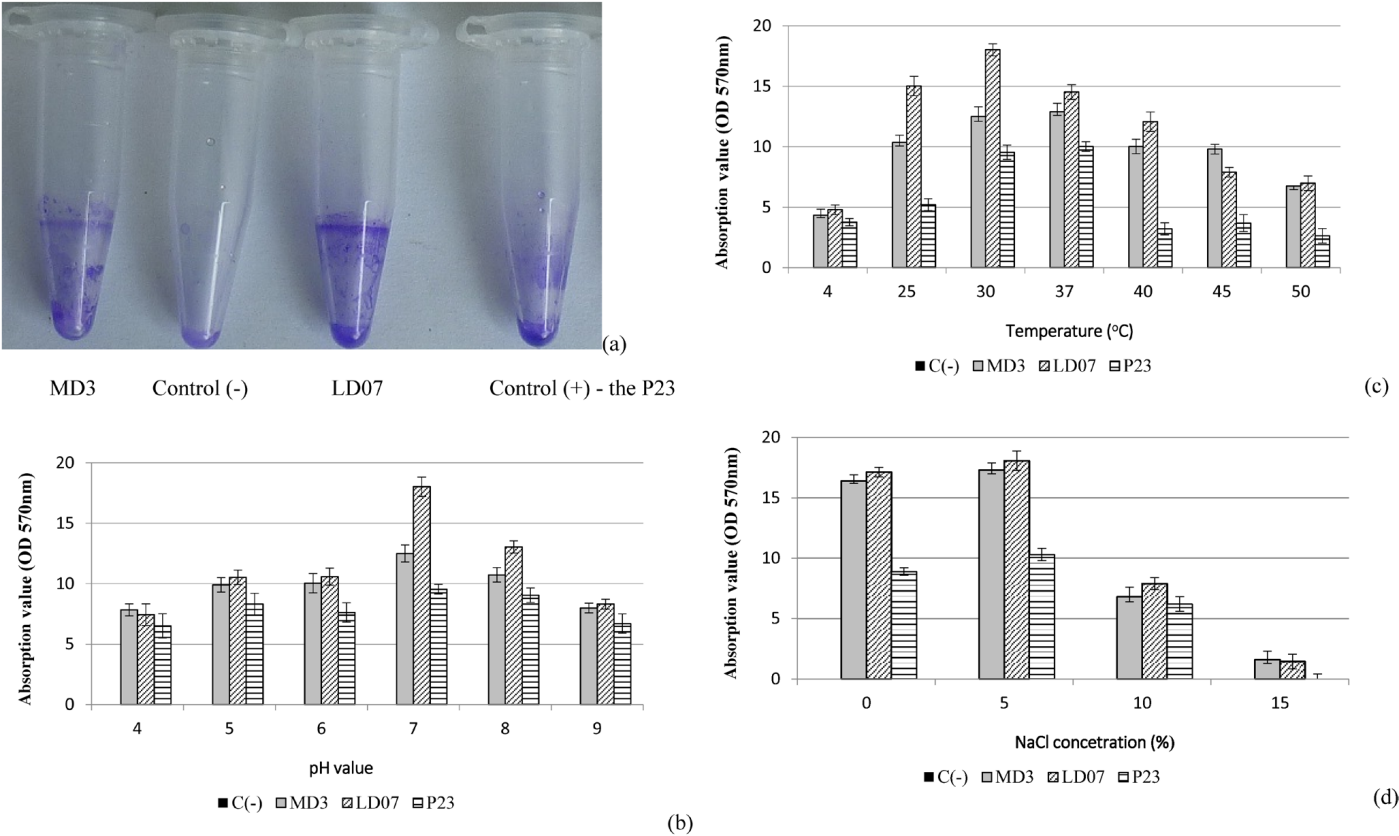
Biofilm formation of the isolated strains MD3 and LD07 in comparison with negative control and positive control P23 (a); and the effects of pH values (b), temperature (c) and NaCl concentration (d). Error bar represents the standard deviation (*n* = 3).

The results in Figure 2b indicated that all three strains, including MD3, LD07 and the positive control P23, are good biofilm formers across pH values from 4 to 9. The negative control has no biofilm. The 2 isolates formed better biofilm than the P23 at all pH values. The optimal pH for formation was 7. Figure 2c showed that from 25°C to 45°C, MD3 and LD07 are well‐formed biofilms. However, at 4°C and 50°C, biofilm formation decreased markedly. The positive control, P23, formed biofilm well only at 30°C–37°C. Figure 2d expressed that all three strains formed biofilms well in the presence of NaCl at concentrations from 0% to 5%. At 10%–15%, biofilm formation was not remarkable. These results suggested that the isolated strains MD3 and LD07 could be applied over a wide pH and temperature range, but not at high NaCl concentrations.

### 3.3 Chemical–Physical Characteristics of Sugarcane Bagasse

Before using sugarcane bagasse as a bio‐carrier, its structure was analysed by EDX, BET and XRD.

#### 3.3.1 EDX Result

The EDX spectrum of the sugarcane bagasse (SB) sample exhibits a profile characteristic of lignocellulosic biomass, predominantly composed of carbon and oxygen (Figure 3a). The SEM image of SB was presented in Figure 3b. Quantitative analysis reveals that carbon is the primary constituent, accounting for 47.64% by weight and 54.80% by atomic percentage. This elemental dominance corroborates the presence of a robust structural framework consisting of cellulose, hemicellulose and lignin. Complementarily, the oxygen content (52.36 wt%; 45.20 at%) is indicative of the abundant hydroxyl (–OH), carbonyl (C=O) and ether (C–O–C) functional groups typical of plant‐derived fibres.

**FIGURE 3:**
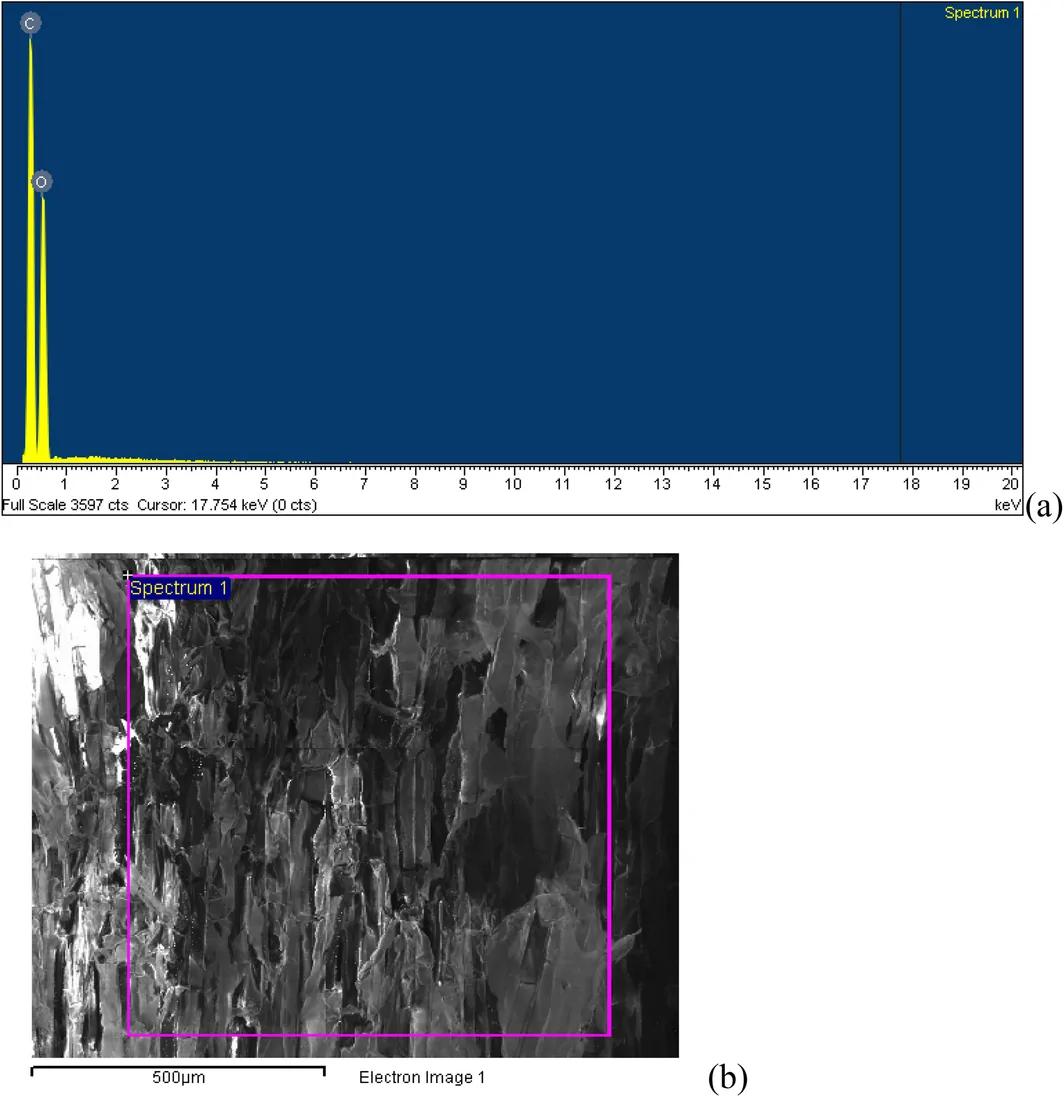
EDX analysis of sugarcane bagasse (a) and SEM image of the material (b).

#### 3.3.2 BET Result

The nitrogen adsorption–desorption analysis provides critical insights into the structural characteristics of raw SB. The Brunauer–Emmett–Teller (BET) specific surface area was measured at 1.2916 m^2^/g, a value consistent with unmodified lignocellulosic biomass which typically exhibits limited surface area prior to chemical or thermal activation. According to the Barrett–Joyner–Halenda (BJH) and Dollimore–Heal (D–H) models, the average pore width was 2.0781 nm. Following IUPAC nomenclature, this classifies the material at the lower threshold of mesoporosity, bordering the microporous range (< 2 nm). Furthermore, t‐plot analysis indicates that the external surface area (1.1118 m^2^/g) constitutes the vast majority of the total surface area, while the Horvath‐Kawazoe maximum pore volume remains negligible at 0.000328 cm^3^/g.

The recorded isotherm exhibits an atypical profile, characterized by a decrease in the quantity of gas adsorbed at higher relative pressures (*p*/*p*_0_ > 0.4) and negative desorption values. In the context of high‐precision manometric measurements, such anomalies are frequently attributed to buoyancy effects or thermal instability, particularly when evaluating low‐surface‐area samples (< 10 m^2^/g), where the mass of nitrogen displaced by the sample can interfere with the sensitivity of the pressure transducers. Despite these physical measurement constraints, surface energy distribution analysis reveals a significant concentration between 20 and 30 *e*/*k*.

#### 3.3.3 XRD Result

The XRD pattern of the raw sugarcane bagasse exhibits characteristic peaks consistent with lignocellulosic materials, primarily dominated by the crystalline structure of cellulose I (Figure 4). The XRD analysis of the SB provides critical evidence of its semi‐crystalline nature, which is a fundamental determinant of its performance as a microbial carrier. The diffractogram exhibits a prominent, high‐intensity reflection at approximately 2*θ* ≈ 22.5°, corresponding to the (200) crystallographic plane of cellulose I. Additionally, a secondary, broad shoulder is observed between 15° and 18°, representing the overlapping (110) and (1−10) planes. These crystalline features are superimposed upon a broad amorphous “hump,” signifying a substantial fraction of disordered components, primarily hemicellulose and lignin, which lack long‐range molecular order. This crystalline‐amorphous duality, characterized by a moderate Crystallinity Index (CrI), is essential for maintaining the mechanical integrity of the fibres within complex soil matrices.

**FIGURE 4:**
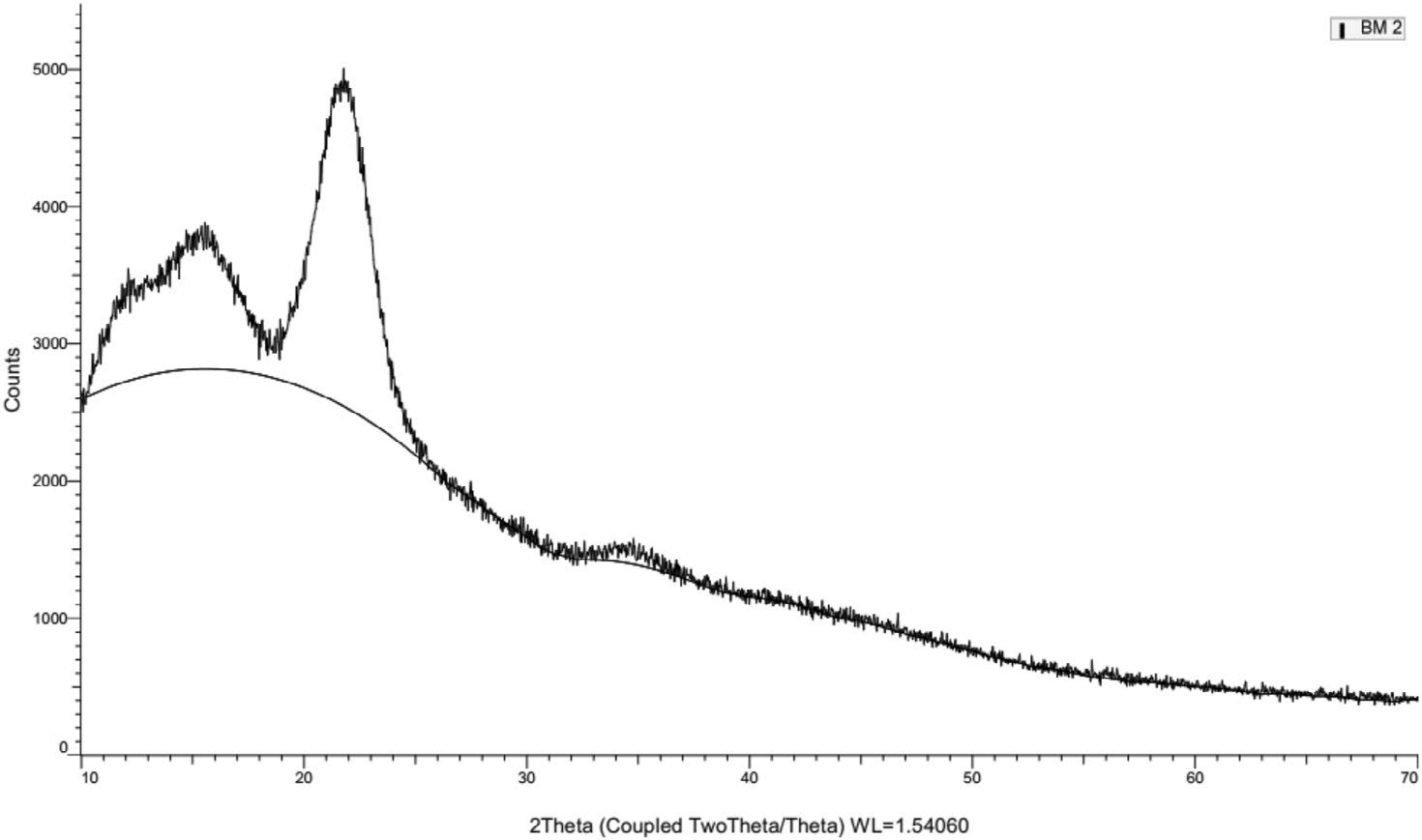
XRD analysis of sugarcane bagasse.

### 3.4 Chlorpyrifos Biodegradation

Using 2 isolated strains, MD3 and LD07, to ferment on sugarcane bagasse, the bio‐product was created. Different concentrations of the bio‐product, including 1%, 5%, 10% and 25% were incubated with different concentrations of CP (Table 1). The amount of remaining CP after 14 and 35 days was analysed by GC–MS/SIM. The CP degradation efficiency was presented in Figure 5.

**FIGURE 5:**
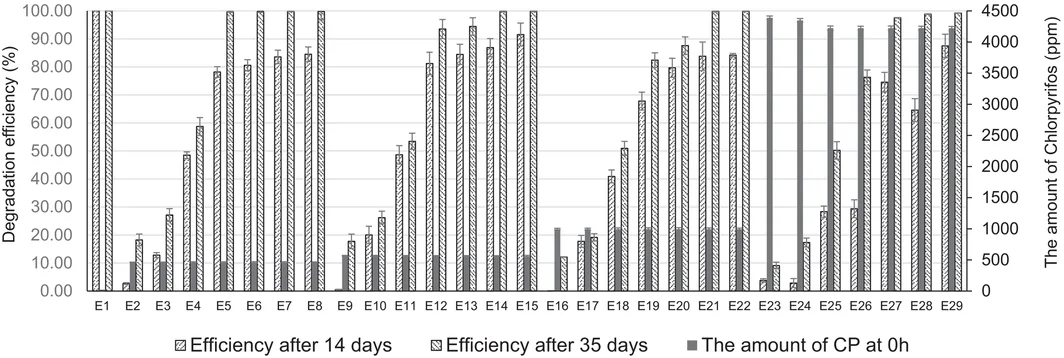
Efficiency after 14 and 35 day incubation with different concentrations of CP (see Table 1). Error bar represents the standard deviation (*n* = 3).

The results in Figure 5 indicated that, initially, there was a small amount of CP (about 11 ppm) in coffee‐growing soil, but after 14 days, the CP had disappeared. In the same manner, in all experiments containing only soil and CP, a small amount of CP was decreased. The experiments, E3, E10, E17 and E24, included only the carrier and a number of CP were also absorbed (27.12%, 26.20%, 19.16% and 17.32%, respectively). In contrast, the experiments E4, E11, E18 and E25 were conducted with only bacteria without a carrier; the higher CP amounts (about 58.74%, 53.44%, 50.94% and 50.21% CP) were degraded. The experiments—E5, E12, E19 and E26—with 1% bio‐product could remove 99.67%, 93.53%, 87.62% and 76.31% of CP at initial concentrations of 460, 570, 1000 and 4500 ppm after 35 days. Meanwhile, more than 94% of CP at different concentrations was degraded after 35 days when 5%, 10% and 25% of the bio‐product were supplemented. Then, 5% of the bio‐product could be used to remove CP residues from coffee‐growing soil. Significant differences among treatments became more pronounced during the later stages of incubation (*p* < 0.05).

To confirm the presence of MD3 and LD07 on the sugarcane bagasse, SEM was taken (Figure 6). In Figure 6a, the control, which was the sugarcane bagasse without bacteria, was observed, but in Figure 6b, bacterial cells appeared. Subsequent to enrichment, isolation and selection, strains MD3 and LD07 were subjected to SEM to evaluate their cellular morphology (data not shown). Guided by prior single‐strain SEM micrographs, strains MD3 and LD07 were identified as illustrated in Figure 6b.

**FIGURE 6:**
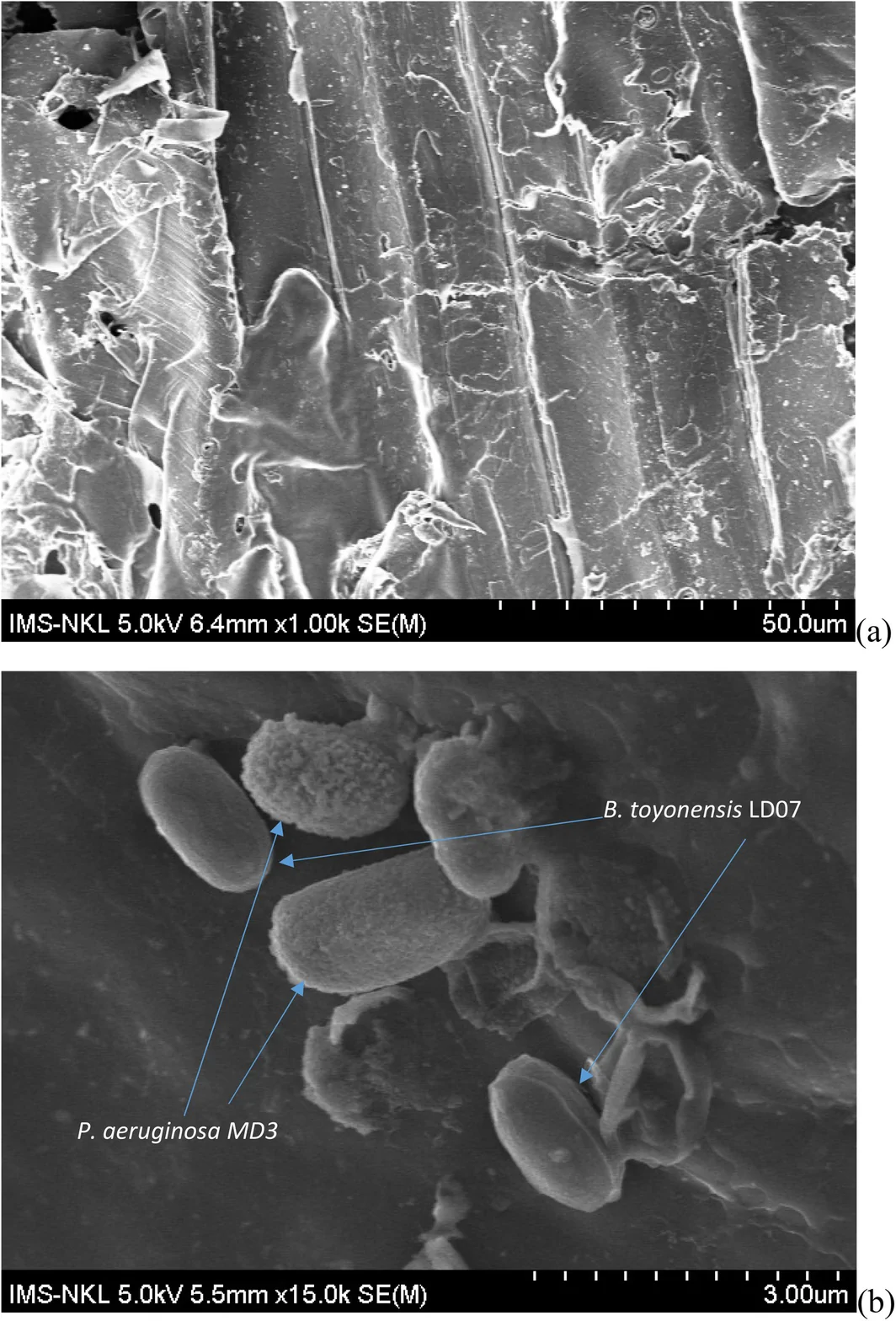
Scanning electron microscopy images of bagasse (a); and bacteria immobilized on bagasse (b).

### 3.5 16S rRNA Gene Amplicon Sequencing

The DNA samples must meet the requisite concentration, yield and purity standards for downstream library preparation. The results pertaining to the quantification and purity assessment of the samples are summarized in Table 2. The results shown in Table 2 also indicate that the concentrations of the gene library in each sample were sufficient for Next Generation Sequencing analysis. Moreover, sequencing errors can significantly compromise the accuracy of downstream bioinformatic analyses. To obtain high‐quality reads for subsequent investigations, raw sequencing data must undergo processing to remove ambiguous bases (N), adapter sequences and low‐quality bases at both the 5′ and 3′ termini (Table 2).

**TABLE 2:** Quality metrics of DNA samples, gene library and sequence reads.

Quality metrics of DNA samples					
Sample	DNA concentration (ng/μL)	OD260/OD280	Amount (ng)	QC result	Fastq_file
M0	114.73	1.81	5163.00	Passed	RO1822412A04
M1	10.53	1.99	473.70	Passed	RO1822412A01
M3	14.40	1.70	648.00	Passed	RO1822412A03

Quality metrics of gene libraries			
Sample	Gene library concentration (nM)	QC result	Note
M0	44.80	Passed	
M1	100.00	Passed	
M3	66.00	Passed	

Quality metrics of sequence reads					
Fastq_file	Total sequence	Total base	Sequence length average	Sequence quality average	% > Q10
RO1822412A04	57,698	90,188,377	1563.10	11.60	81.8%
RO1822412A04‐passed	55,429	87,540,908	1579.30	11.70	82.4%
RO1822412A01	75,236	105,928,618	1408.00	11.60	80.8%
RO1822412A01‐passed	68,609	101,485,380	1479.20	11.70	81.9%
RO1822412A03	77,801	121,447,451	1561.00	11.70	82.5%
RO1822412A03‐passed	75,234	118,847,768	1579.70	11.80	83.0%

*Note:* Samples are processed as paired sets. For instance, Sample A represents the raw sequencing data, while Sample A‐passed denotes the sample after low‐quality reads are removed (post‐quality filtering). (1) Total Bases: The aggregate number of bases sequenced for each sample. (2) % > Q10: The percentage of bases achieving a Phred quality score (Q‐score) greater than 10 relatives to the total sequenced bases.

The number of phylum, class, order, family, genus and species in each sample was presented in Table 3. The results provided that the amount of phylum, class, order and genus in M0—coffee‐growing soil and M1—coffee‐growing soil supplemented with 4500 ppm CP was higher than in M3—coffee‐growing soil supplemented with 4500 ppm CP and 10% bio‐product; the number of family and species in M1 and M3 was higher than in M0. 16S rRNA gene amplicon sequencing of coffee‐growing soils across different treatments (M0, M1 and M3) was deposited in NCBI for Sequence Read Archive (SRA) with accession number PRJNA1529359.

**TABLE 3:** The number of identified phylum, class, order, family, genus and species in three samples by 16S rRNA gene amplicon sequencing.

	Total	M0	M1	M3
Phylum	23	21	20	15
Class	73	63	61	47
Order	124	101	103	77
Family	215	77	82	148
Genus	442	318	337	294
Species	936	568	667	611

#### 3.5.1 Alpha Diversity

Alpha diversity metrics describe the richness and evenness of microbial species within each specific sample (Figure 7). M1 exhibits the highest microbial richness with 754 unique taxa; M3 follows with 698 unique taxa; M0 has the lowest richness at 655 unique taxa (Figure 7a,b). The addition of CP in M1 and M3 appears to increase the overall number of detectable microbial species compared with untreated soil (M0). The Shannon index, which indicates microbial diversity, showed that M1 also had the highest overall diversity and evenness (8.31); M0 was slightly lower at 8.18, while M3 had the lowest (7.43). Although M3 has more species than M0, its lower Shannon index suggests that a few taxa have become highly dominant (likely due to the high 10% chlorpyrifos concentration), reducing the community's overall evenness (Figure 7c). As illustrated in the Figure 7d, the Simpson diversity index was evaluated across three samples including M0, M1 and M3. Overall, all samples maintained relatively high index values exceeding 0.975. Specifically, model M1 achieved the highest performance, peaking slightly above 0.992, closely followed by M0 at approximately 0.9918. In contrast, model M3 exhibited a noticeable decline, dropping to roughly 0.9798. This marked discrepancy highlights that while M0 and M1 demonstrate comparable efficacy with marginal variance, M3 exhibits a significant reduction in Simpson index performance under identical evaluation conditions.

**FIGURE 7:**
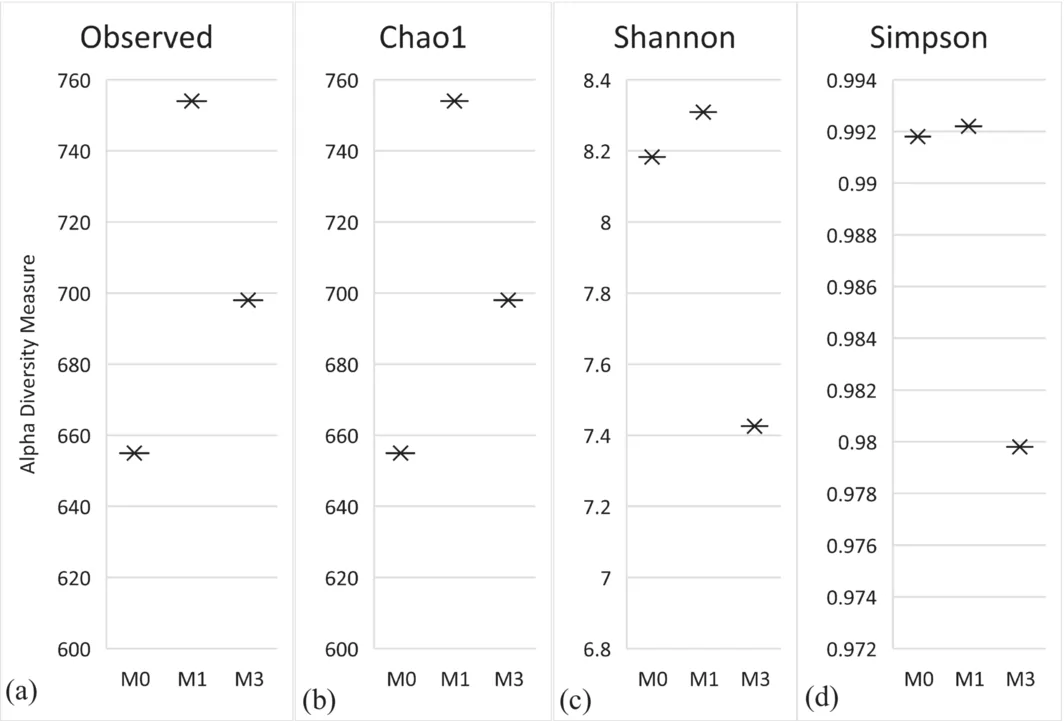
Alpha diversity of bacterial species in three soil samples. Analysis of alpha diversity using several indices, such as Observed (a), Chao 1 (b), Shannon (b) and Simpson index (d). The first graph shows the total number of observed species across the samples.

Taxonomic composition at phylum, class, genus and species levels informed that the microbial community structure shifts significantly across the three treatments (Figure 8a–d, respectively). At phylum level, in M0 sample, Pseudomonadota, Acidobacteriota and Planctomycetota were dominated; but M1 showed a substantial increase in Bacillota (formerly Firmicutes) and Pseudomonadota; meanwhile in M3, a massive dominance of Bacillota was characterized, which occupies nearly 50% of the relative abundance. Then, the bio‐product and chlorpyrifos treatment promote the expansion of *Bacillota*, particularly under high‐stress pesticide conditions (M3).

**FIGURE 8:**
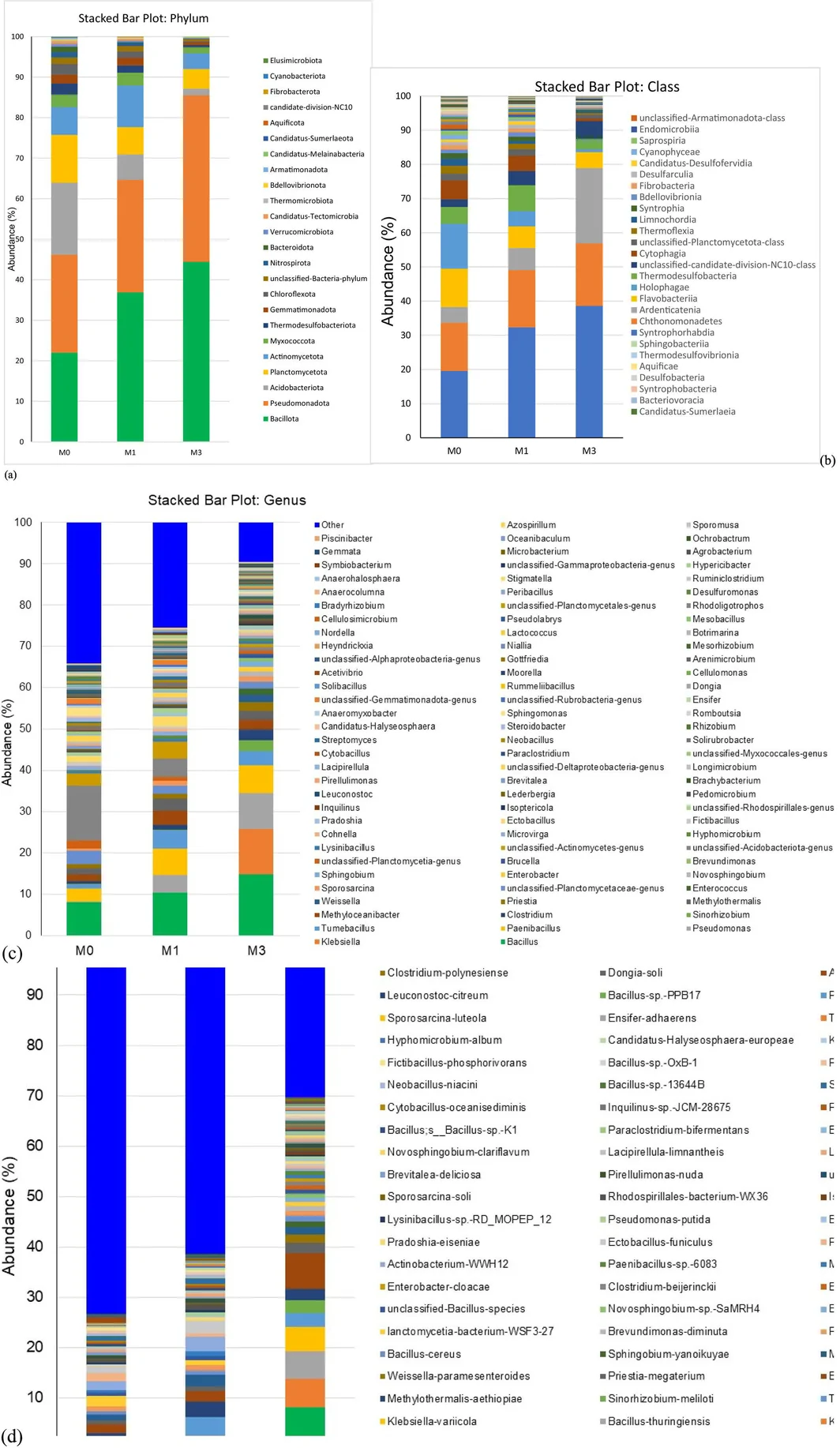
Phylum, class, genus and species composition of bacteria in M0, M1 and M3 samples. Phylum (a), class (b), genus (c) and species (d) relative abundances were illustrated across samples. M0, coffee‐growing soil; M1, coffee‐growing soil supplemented with 4500 ppm CP; and M3, coffee‐growing soil supplemented with 4500 ppm CP and 10% bio‐product.

At class level, the impact of CP stress is expressed in sample M0 and M1. In detail, the introduction of 4500 ppm chlorpyrifos in M1 induced a clear taxonomic shift compared to the M0 baseline (Figure 8b). Syntrophorhabdia expanded. There is a notable increase in the relative abundance of *Syntrophorhabdia* (blue) in M1 compared to M0, suggesting these taxa may possess an intrinsic tolerance to high concentrations of organophosphate pesticides. The diversity was reduced. The “rare biosphere” (represented by the thinner bands at the tops of the bars) appears more compressed in M1, suggesting that high pesticide stress may have suppressed sensitive indigenous taxa, thereby decreasing alpha diversity. Bio‐augmentation and carrier amendment in samples M1 and M3 affected the diversity. The application of the microbial consortium ( *P. aeruginosa* MD3 and *B. toyonensis* LD07) immobilized on sugarcane bagasse in M3 resulted in a distinct restructuring of the community. Ardenticatenia and Chthonomonadetes were dominant. Sample M3 shows a substantial proliferation of *Ardenticatenia* (grey) and *Chthonomonadetes* (orange). This shift likely reflects the successful colonization of the added consortium or the biostimulation of specific indigenous functional groups facilitated by the sugarcane bagasse carrier. The relative abundance of *Flavobacteriia* (yellow) and *Holophagae* (light blue), which were prominent in M0 and M1, significantly decreased in M3. This suggests a competitive displacement by the introduced bio‐cleansing agent and its associated carrier. A visible increase in *Endomicrobiia* (dark blue) is observed in M3, which may contribute to the complex metabolic pathways required for the complete mineralization of chlorpyrifos metabolites.

The transition from M1 to M3 demonstrates that the 10% bio‐product amendment effectively altered the microbial profile of the contaminated soil. The dominance of specific classes in M3 indicates a specialized community potentially optimized for chlorpyrifos biodegradation and soil health recovery. This taxonomic restructuring supports the efficacy of cell immobilization on agricultural by‐products such as sugarcane bagasse to enhance the survival and functional activity of degrading strains in pesticide‐polluted environments.

At genus level, the results in M0 sample confirmed that features a balanced distribution of *Bacillus*, *Pseudomonas* and various unclassified *Acidobacteriota* and *Planctomycetaceae* (Figure 8c). There is a notable presence of *Bacillus*, *Paenibacillus* and *Tumebacillus* in M1 sample. And the results in M3 displayed high dominance of *Bacillus* and *Klebsiella*. Members of the *Bacillus* genus are likely the primary active components of the 10% bio‐product, as they remain dominant even at very high chlorpyrifos concentrations.

At species and sub‐species identification, the full‐length 16S sequencing allows for high‐resolution identification of specific degrading candidates (Figure 8d). The key dominant species in M1 and M3 was *P. aeruginosa* . *K. pneumoniae* and *B. toyonensis* was highly abundant in sample M3. The bio‐product was fermented by *P. aeruginosa* MD3 and *B. toyonensis* LD07, which may explain the dominance of these species in sample M3. *P. aeruginosa* and *B. toyonensis* was available in all 3 samples, but the abundance was higher in the M1 sample and the highest in M3. These results explained the presence of two species, *P. aeruginosa* and *B. toyonensis*, during an enrichment experiment and confirmed their CP‐degrading capacity. *Bacillus thuringiensis* and *Bacillus cereus* were prominent in both M1 and M3 samples, likely contributing to the survival and degradation processes in contaminated soil. *Tumebacillus avium* and *Priestia megaterium* were identified within the community, often associated with soil health and bioremediation. Control Soil (M0) showed higher relative abundances of specialized soil bacteria, including *Sinorhizobium meliloti* , *Methylothermalis aethiopiae* and various unclassified *Acidobacteriota* species. These appear to be suppressed in the M3 sample.

In general, Sankey diagrams representing the hierarchical taxonomic distribution of the soil microbial community from domain to species level of all 3 samples (M0, M1 and M3) were presented in Figure 9.

**FIGURE 9:**
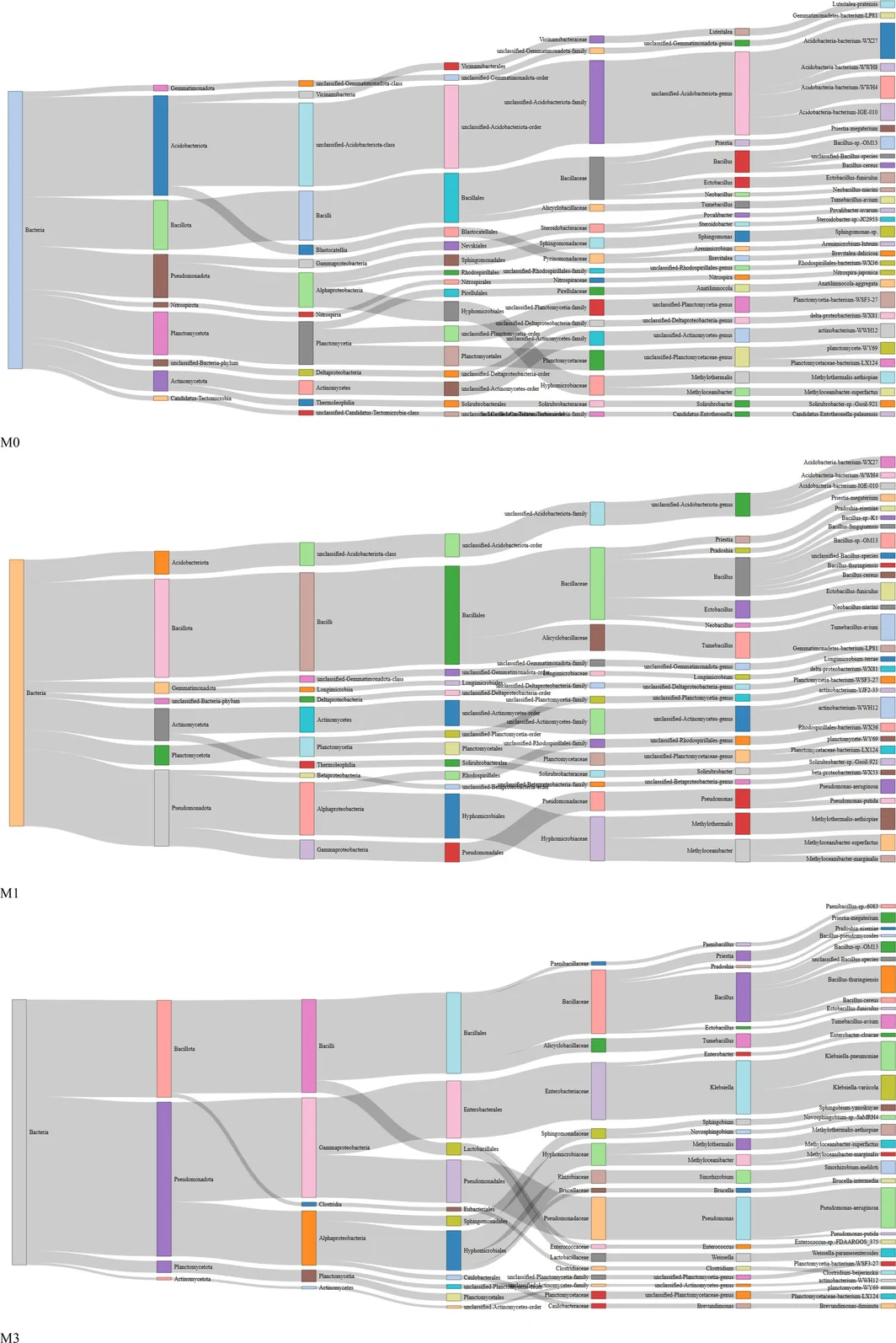
Sankey diagram representing the hierarchical taxonomic distribution of the soil microbial community from domain to species level.

#### 3.5.2 Beta Diversity

Beta diversity represents the variations in microbial composition and distribution across different samples or environments. In 16S rRNA gene amplicon sequencing studies, beta diversity is used to characterize differences between biological groups or experimental treatments. These metrics are computed using distance‐based metrics; in this report, beta diversity was determined using the Bray–Curtis dissimilarity index (Figure 10). This index estimates the disparity in the presence and abundance of microorganisms for each sample pair. The values range from 0 to 1, where 0 signifies no variance (identical composition) between the compared pair.

**FIGURE 10:**
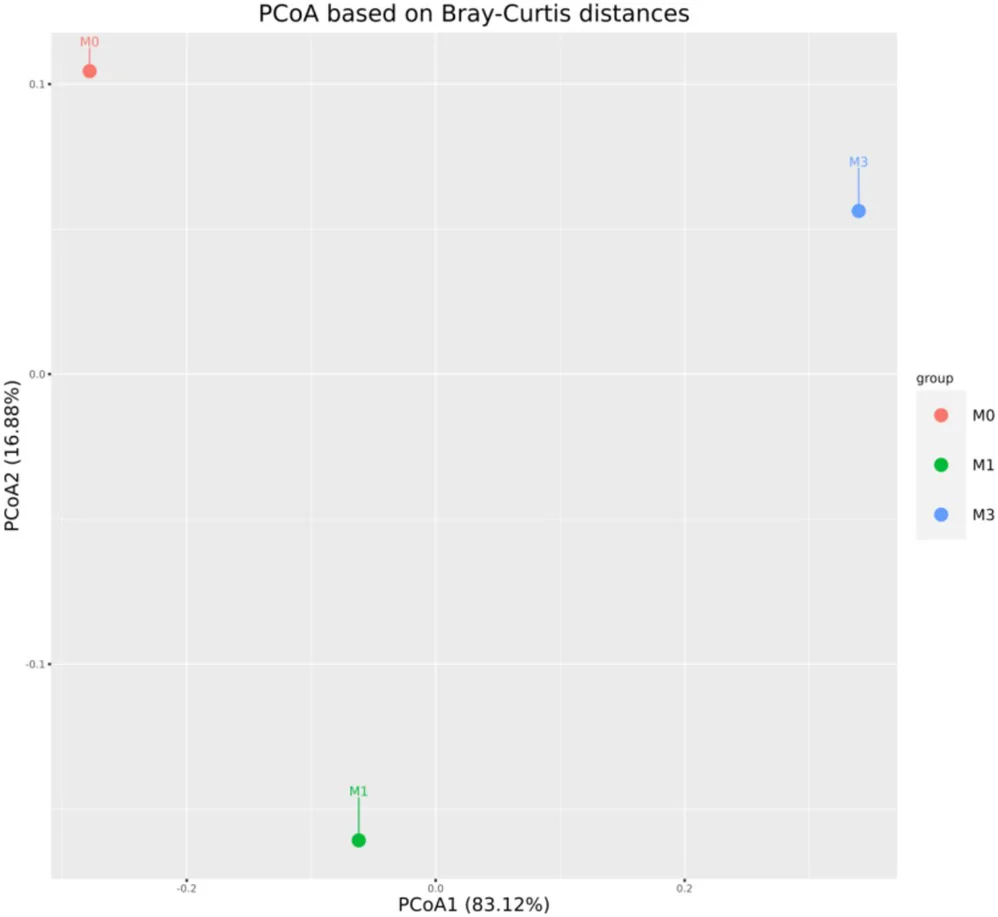
PCoA plot generated based on the Bray‐Curtis distance matrix. Samples belonging to the same group are represented by consistent colours (Group 1) or symbols (Group 2). In each plot, the vertical and horizontal axes correspond to the two principal coordinate axes that capture the maximum variance between sample points. The percentage values displayed on each axis indicate the proportion of variance explained by those coordinates regarding the overall dissimilarity among samples.

To capture inter‐sample distances (degree of dissimilarity) in large‐scale datasets, visualization tools such as multidimensional scaling (MDS) are essential. Principal Coordinate Analysis (PCoA) is a classical form of MDS, utilized as an ordination method to map data points in a multidimensional space. The spatial proximity between sample points reflects their degree of similarity; samples positioned closer together exhibit higher compositional similarity, whereas those with analogous microbial profiles tend to form distinct clusters. As illustrated in Figure 10, the dissimilarity of the investigated samples was evaluated using the Bray‐Curtis index.

The PCoA plot shows that M0, M1 and M3 are clearly separated into distinct clusters. PCoA1 (83.12%): This primary axis explains the vast majority of the variation, placing M0 at one extreme and M3 at the other. This indicates that the combination of the bio‐product and chlorpyrifos concentration has a profound and distinct impact on the microbiome diversity in soil, fundamentally altering the microbial community structure from its original state.

## 4 Discussion

### 4.1 Isolation of Chlorpyrifos‐Degrading Bacteria

The enrichment strategy successfully recovered two chlorpyrifos‐degrading bacteria, identified as *Pseudomonas aeruginosa* MD3 and *Bacillus toyonensis* LD07. Both *Pseudomonas* sp. and *Bacillus* sp. are frequently reported as effective degraders of organophosphate pesticides owing to their broad metabolic capabilities and adaptability to contaminated environments (Mandree et al. 2021; Mishra et al. 2021; Liu et al. 2016; Ahirwar et al. 2019; Maharana et al. 2025). *Pseudomonas* sp.—including *P. aeruginosa, P. syringae*, *P. putida* and *P. stutzeri* —efficiently catabolize CP through organophosphate hydrolase (OPH) and dehalogenase enzymatic pathways, which are frequently coupled with the complete mineralization of 3,5,6‐trichloro‐2‐pyridinol (TCP) (Lu et al. 2020; Aswani et al. 2019; Zhu et al. 2019; Alidoosti et al. 2024). *Bacillus* sp. such as *B. toyonensis*, *B. cereus* , *B. subtilis* and *B. megaterium* , are reported to hydrolyze CP and TCP (Bose et al. 2021; Wepukhulu et al. 2024; Bala et al. 2022). Asamba et al. (2022) isolated 18 CP degrading bacterial strains which belong to *Pseudomonas*, *Stenotrophomonas*, *Bacillus*, *Alicaligenes* and *Achromobacter* species. By using 16S rRNA gene sequencing, Ahirwar et al. (2019) estimated that *B. megaterium* , *B. soli* strain R, *B. drentensis* , *B. koreensis* and *Microbacterium paraoxydans* represented 8% of total bacteria in CP contaminated soil sample. Their successful isolation from agricultural soil in the present study suggests that long‐term pesticide application may have selected indigenous microbial populations capable of utilizing chlorpyrifos as a substrate. However, the above publications did not mention on biofilm type in CP‐degrading experiments and to our knowledge, the articles on *B*. *toyonensis* are still limited. The amount of CP in our investigation (460, 575, 1000 and 4500 ppm) was much higher than in those documents (< 300 ppm) and the removal efficiency of the *P. aeruginosa* MD3 and *B. toyonensis* LD07 was remarkable.

The use of glucose as a co‐substrate during enrichment likely promoted initial bacterial growth and increased the probability of recovering microorganisms capable of co‐metabolizing chlorpyrifos. Similar enrichment strategies have been widely used to isolate pesticide‐degrading bacteria because an additional carbon source supports cell growth before the target contaminant can be efficiently transformed (Du et al. 2024; Zhai et al. 2024; Li et al. 2021). *Bacillus cereus* CP6 and *Klebsiella pneumoniae* CP19 actively degrade chlorpyrifos (> 70% at 200–300 ppm), with strain CP19 displaying superior degradation kinetics under submerged conditions (Elshikh et al. 2022). Biodegradation for both strains was optimized at neutral pH in the presence of glucose, though their preferred organic nitrogen sources differed—peptone for *B. cereus* CP6 and beef extract for *K. pneumoniae* CP19. Co‐inoculation of both strains enhanced removal efficiency synergistically, achieving 93.4% ± 2.8% degradation in liquid culture and up to 94.5% ± 3.3% in bioaugmented soil over 16 days. These isolates therefore provide suitable candidates for developing bioaugmentation products for pesticide‐contaminated soils.

### 4.2 Biofilm Formation and Suitability of Sugarcane Bagasse

Both isolates formed stable biofilms under laboratory conditions, although *B. toyonensis* LD07 consistently produced greater biofilm biomass than *P. aeruginosa* MD3. Biofilm formation is considered an important adaptive strategy because surface‐associated cells generally exhibit greater tolerance to environmental stress than planktonic cells. The extracellular polymeric substances (EPS) surrounding biofilm cells enhance cell adhesion, facilitate nutrient retention and protect microorganisms from adverse environmental conditions (Nadar and Khan 2024).

The physicochemical analyses further supported the suitability of sugarcane bagasse as an immobilization carrier. SEM images confirmed successful bacterial attachment and biofilm development on the carrier surface, while BET analysis indicated partial occupation of the pore structure following bacterial colonization. In contrast, XRD analysis showed that bacterial immobilization did not alter the crystalline structure of the lignocellulosic material, suggesting that the carrier maintained its structural stability throughout biofilm formation. Similar observations have been reported for other lignocellulosic carriers used in environmental bioremediation (Tao et al. 2015; Sarker et al. 2017; Varghese et al. 2022). Together, these findings demonstrate that sugarcane bagasse provides a suitable physical support for bacterial immobilization while preserving its structural integrity (Sakhaem et al. 2026; Soliman et al. 2024; Machado and Mulky 2023; Khalil et al. 2022).

### 4.3 Enhanced Chlorpyrifos Degradation by Immobilization

The immobilized bio‐product consistently outperformed free bacterial cells during chlorpyrifos degradation in the soil microcosm experiment. This improvement is likely associated with the establishment of stable biofilms on the carrier surface, which enhance bacterial persistence and maintain higher cell density throughout the degradation process. Immobilization also minimizes cell loss and improves resistance to environmental fluctuations, thereby prolonging microbial activity in soil (Ahirwar et al. 2019; Biswal and Malik 2022; Sridharan and Krishnaswamy 2022).

Sugarcane bagasse may have provided additional benefits beyond physical support. As a lignocellulosic material rich in cellulose and hemicellulose, it can serve as an auxiliary carbon source that sustains microbial metabolism during cometabolic degradation. Similar improvements in pesticide degradation following bacterial immobilization have been reported using various natural carrier materials, although agricultural residues remain particularly attractive because of their low cost, high availability and environmental sustainability (Fuentes et al. 2013; John et al. 2016; Abdelhafez and Li 2016; Al‐Amshawee et al. 2020; Jagaba et al. 2024). These characteristics support the potential application of the developed bio‐product for in situ remediation of chlorpyrifos‐contaminated soils.

### 4.4 Response of the Indigenous Soil Bacterium Community

Amplicon sequencing demonstrated that bioaugmentation influenced the composition of the indigenous bacterial community throughout the remediation process. The increased abundance of *Bacillus* and *Pseudomonas* following inoculation was expected because these genera represented the introduced degraders. More importantly, the overall community structure also shifted, indicating that bioaugmentation affected not only the inoculated strains but also the surrounding microbial populations.

Changes in bacterial community composition during pesticide degradation have been widely reported and are generally associated with alterations in nutrient availability, intermediate metabolites and ecological interactions among microorganisms (Maharana et al. 2025; Pham et al. 2024; Bhende et al. 2022). The observed community shifts therefore suggest that chlorpyrifos degradation was accompanied by microbial succession rather than simple replacement of the native microbiota. Such ecological responses are important because they indicate that successful bioremediation depends on interactions between introduced degraders and indigenous microorganisms.

The comparative analysis of bacterial community composition revealed a pronounced taxonomic succession across the experimental treatments from the baseline condition (M0) to enriched/treated states (M1 and M3), spanning the levels of *phylum, class, order, family, genus and species* (Figure 8). While the initial microbiota (M0) was characterized by higher diversity and equitability, dominated by slow‐growing and oligotrophic taxa, selective pressure or biostimulation/bioaugmentation noticeably shifted the microbial consortium toward functional degraders capable of utilizing xenobiotic compounds.

At the *phylum and class levels*, the relative abundance of *Firmicutes* (predominantly belonging to the class *Bacilli*) and *Pseudomonadota* (formerly *Proteobacteria*, notably *Gammaproteobacteria* and *Alphaproteobacteria*) exhibited substantial enrichment from M0 to M1 and M3, while secondary phyla such as *Planctomycetota*, *Actinomycetota* and *Gemmatimonadota* were progressively outcompeted. These findings are in strong agreement with recent metagenomic surveys demonstrating that exposure to organophosphate pesticides, particularly chlorpyrifos, exerts strong selective pressure favouring *Firmicutes* and *Pseudomonadota* due to their intrinsic metabolic plasticity, membrane resilience against organophosphate toxicity and repertoire of hydrolytic enzymes (such as organophosphate hydrolases [OPH] and phosphotriesterases [PTE]) (Kumar et al. 2022, 2023).

At the *order and family levels*, this ecological transition was manifested by a notable proliferation of members within *Bacillales* (family *Bacillaceae*), *Pseudomonadales* (family *Pseudomonadaceae*) and *Enterobacterales* (family *Enterobacteriaceae*), displacing environmental soil‐dwelling families such as *Planctomycetaceae* and *Hyphomicrobiaceae*. The preferential enrichment of *Bacillaceae* and *Enterobacteriaceae* reflects their metabolic opportunism and capacity to utilize chlorpyrifos as a sole carbon and phosphorus source, consistent with recent literature identifying *Bacillales* as primary responders in pesticide‐contaminated microenvironments (Elshikh et al. 2022; Anjana and Priya 2026).

At the *genus level* (Figure 8c), striking divergence was observed among the treatments. In M0, the bacterial flora was dispersed among unclassified genera, *Lactococcus*, *Heyndrickxia* and *Sporosarcina*. In contrast, sample M1 demonstrated prominent enrichment of the genus *Bacillus* (~10%–12%) alongside significant proportions of *Enterobacter*, *Clostridium* and *Pseudomonas*. More conspicuously, sample M3 exhibited a substantial co‐enrichment of *Klebsiella* (reaching > 10%) and *Bacillus* (~15%), along with active degrader genera including *Pseudomonas*, *Sphingobium* and *Paenibacillus*. The co‐existence and simultaneous proliferation of *Klebsiella*, *Bacillus* and *Pseudomonas* in M1 and M3 corroborate recent reports by Kumar et al. (2022) and Kiran et al. (2025), which underscored that effective chlorpyrifos mineralization typically requires synergistic microbial consortia wherein *Pseudomonas* and *Enterobacteriaceae* initiate ester hydrolysis into TCP, while *Bacillus* and *Sphingobium* species further catalyse ring cleavage and detoxicative degradation.

At the *species level* (Figure 8d), high‐resolution taxonomic assignment unravelled distinct functional degraders driving the community restructuring: *Sample M1* was uniquely characterized by high relative abundances of *Pseudomonas aeruginosa* , *Brevundimonas diminuta* and *Sphingobium yanoikuyae* , alongside endospore‐formers such as *Bacillus cereus* , *Bacillus thuringiensis* and *Paenibacillus* sp. The involvement of *Pseudomonas aeruginosa* and *Brevundimonas diminuta* is well‐documented in modern organophosphate bioremediation, as they harbour phosphotriesterase‐encoding genes (*opd*/*oph*) that rapidly hydrolyze the phosphoester bond of chlorpyrifos (Bhende et al. 2022). *Sample M3* showed an unmistakable dominance of *Klebsiella pneumoniae* , *Klebsiella variicola* and *Bacillus toyonensis/Bacillus cereus*. Recent investigations by Elshikh et al. (2022) highlighted that *Bacillus cereus* and *Klebsiella pneumoniae* isolated from organophosphate‐rich systems exhibit superior chlorpyrifos biodegradation efficiency (exceeding 70%–80% residue removal within short incubation intervals) via complementary co‐metabolic networks. Furthermore, the persistence of auxiliary species such as *Enterobacter cloacae* , *Pseudomonas putida* and *Priestia megaterium* across M1 and M3 suggests an ecologically resilient multi‐species framework capable of surviving pesticide shock loads while mitigating TCP‐mediated toxicity (Kumar et al. 2022; Ghorab et al. 2026).

Collectively, the taxonomic shifts from M0 to M1 and M3 substantiate the hypothesis that the treatment conditions effectively drove the positive selection and proliferation of specialized chlorpyrifos‐degrading bacterial taxa across all taxonomic tiers.

### 4.5 Environmental Implications

This study demonstrates that combining chlorpyrifos‐degrading bacteria with an inexpensive agricultural by‐product can improve the efficiency of microbial bioaugmentation while simultaneously influencing the structure of the resident soil microbiome. Unlike many previous studies that focused primarily on degradation efficiency, the present work integrates bacterial isolation, biofilm‐based immobilization, carrier characterization, soil microcosm evaluation and microbial community analysis within a single remediation system.

From a practical perspective, sugarcane bagasse represents a renewable and low‐cost carrier that could facilitate the development of environmentally sustainable bio‐products for pesticide‐contaminated soils. Future studies should evaluate the long‐term stability of the immobilized bio‐product under field conditions and investigate the functional responses of the soil microbiome using metagenomic or metatranscriptomic approaches to better understand the mechanisms underlying enhanced chlorpyrifos degradation. The protracted application of chlorpyrifos and related organophosphorus pesticides in agricultural and horticultural practices has led to widespread environmental contamination. Due to their inherent toxicity and relative recalcitrance to natural degradation, these compounds pose a significant threat to both ecological integrity and human health. The utilization of microbial metabolism for bioremediation offers an environmentally benign and economically viable alternative for the degradation of chlorpyrifos (CP) residues (Balakrishnan and Rao 2019).

## 5 Conclusions

This study developed an immobilized bio‐product consisting of two chlorpyrifos‐degrading bacterial strains, *Pseudomonas aeruginosa* MD3 and *Bacillus toyonensis* LD07, using sugarcane bagasse as a low‐cost carrier. The two isolates formed stable biofilms under a wide range of environmental conditions and physicochemical characterization confirmed that sugarcane bagasse provided a suitable surface for bacterial immobilization without altering its structural integrity.

The immobilized bio‐product achieved highly efficient chlorpyrifos degradation in contaminated soil and consistently outperformed free bacterial cells during the microcosm experiment. Amplicon sequencing further demonstrated that bioaugmentation influenced the composition of the indigenous soil bacterial community while supporting chlorpyrifos removal.

Overall, these findings highlight the potential of combining biofilm‐based bacterial immobilization with an agricultural by‐product to develop sustainable bioaugmentation strategies for pesticide‐contaminated soils. This integrated approach provides a practical foundation for future field‐scale applications and contributes to the development of environmentally friendly bioremediation technologies.

## Author Contributions

**Le Thi Nhi‐Cong:** conceptualization, writing – review and editing, project administration, supervision, funding acquisition, software, validation. **Vu Duc Toan:** visualization, investigation, writing – original draft. **Dang Viet Lam:** investigation, writing – original draft, software, methodology, conceptualization, resources. **Tran Thi Mai:** methodology, data curation, investigation, validation. **Cung Thi Ngoc Mai:** data curation, software, formal analysis, validation. **Bui Thi Kim Anh:** validation, formal analysis, data curation. **Dong Diem Linh:** software, formal analysis, investigation.

## Funding

This work was supported by National Foundation for Science and Technology Development (NCUD.01‐2024.03).

## Ethics Statement

This article does not contain any studies with human participants or animals performed by any of the authors.

## Conflicts of Interest

The authors declare no conflicts of interest.

## Data Availability

The data that support the findings of this study are available on request from the corresponding author.
